# Characterization of enteric-coated erythromycin tablets by Raman mapping and its pharmaceutical evaluation

**DOI:** 10.3389/fchem.2023.1270737

**Published:** 2023-10-18

**Authors:** Wenbo Zou, Wanhui Liu, Changqin Hu

**Affiliations:** ^1^ Key Laboratory of Molecular Pharmacology and Drug Evaluation (Yantai University), School of Pharmacy, Ministry of Education, Collaborative Innovation Center of Advanced Drug Delivery System and Biotech Drugs in Universities of Shandong, Yantai University, Yantai, China; ^2^ National Institutes for Food and Drug Control, Beijing, China

**Keywords:** Raman mapping, coating formulation, coating thickness, dissolution, enteric coated tablets

## Abstract

Enteric tablet coating thickness is a critical quality attribute of the coating process that can affect dissolution behavior *in vitro* as well as release *in vivo*. Raman mapping offers unique advantages in analyzing the distribution of active pharmaceutical ingredients and excipients in formulations. In this study, Raman mapping was used to characterize the coating of enteric-coated erythromycin tablets coated by two different processes and compare the differences in their coating formulation, thickness, and uniformity. Furthermore, we aimed to select the appropriate pH of the dissolution medium at which the coating slowly cracks to release the drug and determine the dissolution profile. The differences in the coating thickness and uniformity of the two products resulted in differences in dissolution behavior. Although there are differences in the coating processes for the two types of enteric-coated erythromycin tablets, the thickness of the outer coating on the side is a critical quality attribute in both processes. The outer coating of product A is relatively thick, and the thickness of the outer coating on the side affects the dissolution amount. The outer coating of product B is relatively thin, resulting in a short cracking time and large variation and a significant difference in the initial dissolution amounts between tablets. Raman mapping can be used to analyze the differences in coating formulations and for process evaluation.

## 1 Introduction

Enteric formulations are common formulations characterized by delayed release after oral administration and are suitable for drugs that are acid-unstable or irritate the gastric mucosa. Enteric-coated tablets are made resistant to the acidic environment in the stomach by encapsulation with an enteric coating containing polymeric material that is dissolved and released in the small intestine at a neutral pH (Ahmed et al., 2020; Ahmad, 2022). Controlling the enteric-coated tablet coating process is essential because the coating affects the *in vivo* pharmacokinetic behavior of the drug (Kaunisto et al., 2011; Andersson Trojer et al., 2013). Control of the coating lies primarily in its thickness and uniformity. Analyzing the effect of coating thickness on dissolution and corresponding adjustments can make the enteric-coated tablets conform to specific *in vivo* release requirements. There are various methods for determining coating thickness, including near-infrared spectroscopy (NIRS) (De Beer et al., 2011; Gendre et al., 2011; Avalle et al., 2014; Korasa et al., 2016; Hattori et al., 2018; Wahl et al., 2019; Mohan et al., 2020), Raman spectroscopy (Muller et al., 2012; Wirges et al., 2013; Kim et al., 2016; Barimani and Peter, 2017), optical coherence tomography (Markl et al., 2015a; Markl et al., 2015b; Sacher et al., 2019; Matthias et al., 2022), terahertz pulsed imaging (Brock et al., 2012; Lin et al., 2017; Pei et al., 2018; Dong et al., 2023), X-ray computed tomography (X-CT) (Ariyasu et al., 2017), and multispectral UV imaging (Novikova et al., 2017). Compared with conventional methods of calculating thickness by weight gain after coating, these methods enable direct measurement of coating thickness and can also be used for online control of the coating process.

However, most literature characterizes the thickness of enteric-coated tablets as a whole and does not consider the effect of coating uniformity in different parts of the tablet on its release or the correlation with *in vitro* dissolution behavior. Ariyasu et al. (2017) used NIRS to determine the coating spectra of enteric-coated aspirin tablets from two manufacturers and the coating thickness determined by optical microscopy as a reference value to establish a prediction model to analyze coating thickness and the correlation between the coating thickness of aspirin enteric-coated tablets and the rate of dissolution. In addition, they used X-CT to analyze the homogeneity of the coating of the two preparations. They found that the dissolution rate of products with uniform coating thickness negatively correlated with coating thickness, and there was no correlation between dissolution and coating thickness of the products with inhomogeneous coatings, concluding that the side coating thickness of enteric-coated aspirin tablets was a critical attribute influencing their dissolution rate (Ariyasu et al., 2017).

Raman mapping is a powerful tool for studying the composition of pharmaceutical formulations. Since the area in a formulation in which active pharmaceutical ingredients (APIs) and excipients are located is only a few micrometers in size, information on the physical and chemical characteristics and spatial distribution of these components can be obtained using a microscale confocal Raman spectrometer by scanning the Raman spectra of every point of the surface of the formulation (Dorián László et al., 2022). Raman mapping can be used to analyze the homogeneity of API or excipient distribution in a formulation as well as the particle size distribution to determine the polymorphism of APIs, crystalline transformation, and transparent tablet coating thickness (Moriyama, 2016; Ewing and Kazarian, 2017; Qi et al., 2022).

Erythromycin, a common clinical antibiotic, is acid-unstable and must be formulated as enteric-coated tablets. Unlike enteric-coated aspirin tablets, the coating of enteric-coated erythromycin tablets usually consists of two layers of differently formulated materials. The outer coating is acid-resistant and protects erythromycin from degradation by gastric acid in the stomach. The inner layer between the tablet core and outer coating prevents interaction between the outer coating and API in the tablet core thereby maintaining its stability (Tirpude and Puranik, 2011; Katharina et al., 2022). The relationship between coating thickness and dissolution characteristics of enteric formulations with two coating layers has not been systematically investigated. In the present study, the thickness of the inner and outer coats of enteric-coated erythromycin tablets produced by two different coating processes was characterized by Raman mapping to analyze the differences in coating processes. In addition, the critical quality control points of the formulation coating process were identified by investigating the correlation between the dissolution behavior of the products of different processes and the coating process.

## 2 Materials and methods

### 2.1 Materials

#### 2.1.1 Reagents

The 10 batches of enteric-coated erythromycin tablets used in the present study were approved products from two manufacturers and labeled as products A and B. Six batches were produced by domestic company A. Among these, three samples were 0.25-g formulations designated A-1, A-2, and A-3, and the other three samples were 0.125-g formulations designated A-5, A-6, and A-7. The other four batches were produced by foreign company B and included samples of 0.25-g formulations designated B-1, B-2, B-3, and B-4.

#### 2.1.2 Preparation of solutions

The dissolution medium used for acid resistance testing was 0.1 mol/L hydrochloric acid solution, while that used to determine the dissolution curve was pH 5.5 phosphate buffer (9 mL of 0.2 mol/L sodium hydroxide solution was added to 250 mL of 0.2 mol/L potassium dihydrogen phosphate solution and diluted to 1,000 mL with water).

The dissolution amount was determined using 50 mL of 35 g/L anhydrous dipotassium hydrogen phosphate solution (dilute phosphoric acid adjusted pH to 9.0) as mobile phase A. Next, 400 mL water and 190 mL of tert-butyl alcohol were added, and then 530 mL water was added and mixed (Japanese, 2023).

### 2.2 Methods

#### 2.2.1 Determination of coating thickness by Raman mapping

**FIGURE 1:**
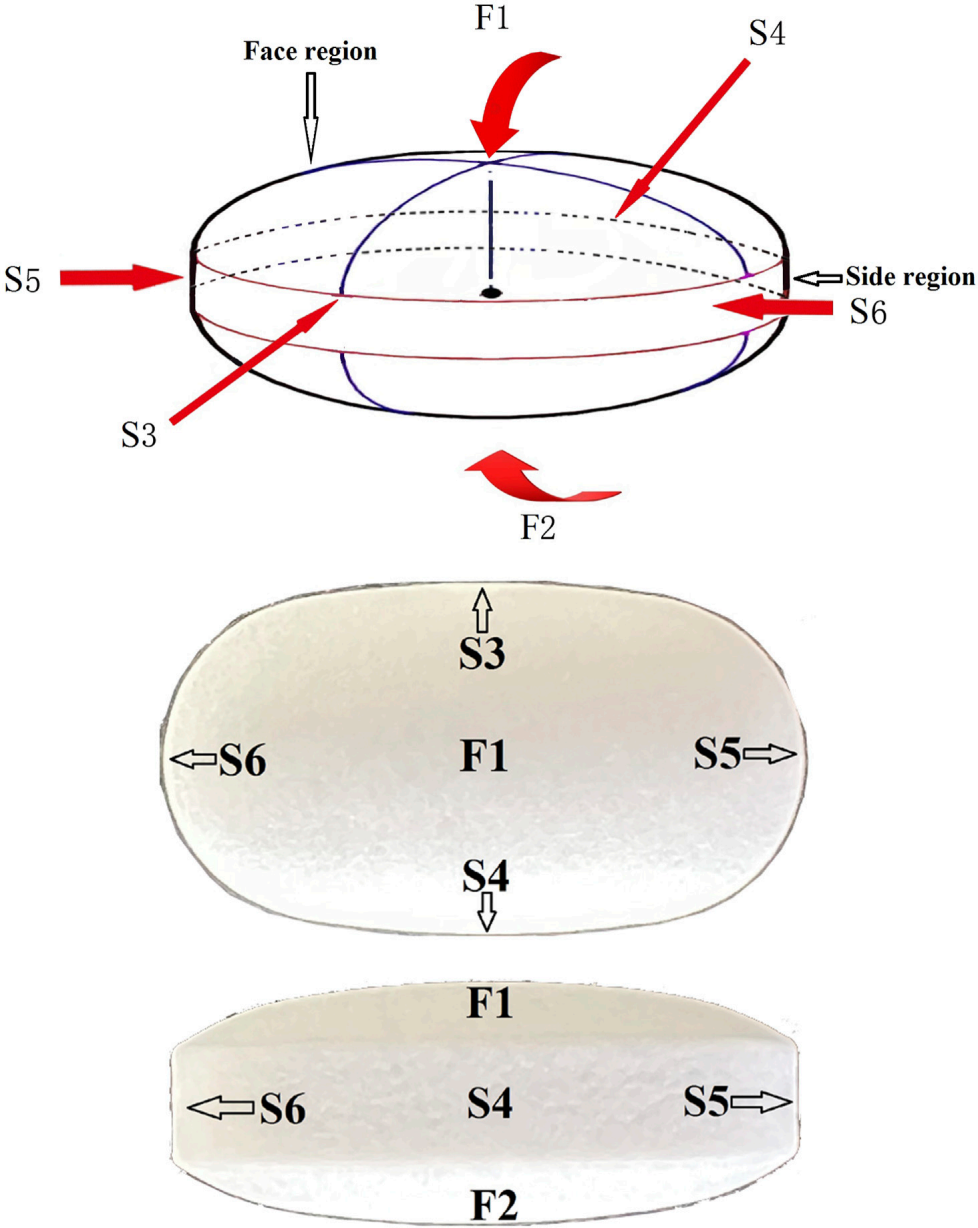
Schematic diagram of enteric-coated erythromycin tablets. The tablets were cut along the blue line. Cross-sectional Raman mapping at the six positions indicated by the arrows was performed to measure coating thickness on the six surfaces.

A Thermo DXR2xi Microscopic Raman Imaging Spectrometer (Thermo Fisher Scientific, Waltham, MA, United States) with a laser assembly (laser, filter, grating), an Olympus microscope system (Olympus Corporation, Tokyo, Japan), a motorized stage, and charge coupled device (CCD) were used. Mapping was performed using a ×50 telephoto objective, a laser wavelength of 532 nm, a laser power of 8.5 mW, an exposure time of 0.2 s, 20 scans, a scanning pixel width of 3.0 μm, and a confocal aperture of 25 μm. The samples were oval tablets (Figure 1). Two sites on the face region and four sites on the side region were selected for a total of six sites to determine the coating thickness of the six sides of the tablets. The two face regions were designated F1 and F2, the side regions at the vertices of the short axis of the ellipse were designated S3 and S4, and the side regions at the vertices of the long axis of the ellipse were designated S5 and S6. Due to the opacity of the sample coating, the laser could not penetrate the coat to make measurements inside the coat; therefore, cross-sectional Raman spectra were collected after cutting the sample along the blue line shown in Figure 1. The spectra were baseline-corrected, imaged, and analyzed using multivariate curve resolution to measure coating thickness. The coating thickness was measured five times on each side, and the average value was calculated as the thickness of the coat on that side. The coatings of six samples per batch were characterized.

#### 2.2.2 Determination of dissolution profile

Six tablets were used from each sample batch, and the dissolution profile of each tablet was determined. We adopted the method outlined in the 2020 Chinese Pharmacopoeia to determine enteric-coated erythromycin tablet dissolution. A SOTAX AT 7X dissolution apparatus (SOTAX, Aesch, Switzerland) was used at a paddle speed of 50 rpm in a water bath at a temperature of 37°C. The samples were first dissolved in 0.1 mol/L hydrochloric acid solution for 60 min and then transferred to pH 5.5 phosphate buffer solution for dissolution. The time at which the coating began to dissolve was designated 0 min, and 5-mL samples were collected at 5, 7.5, 10, 12.5, 15, 17.5, 20, 22.5, and 25 min. After filtration through a 0.45-µm filter membrane, the dissolved amount was determined using a Shimadzu 20AT HPLC instrument (Shimadzu, Kyoto, Japan) with a Waters Xbridgeshiled RP column (4.6 mm × 150 mm, 3.5 µm; Waters Corporation, Milford, MA, United States). The mobile phase was A-acetonitrile (60:40), the column temperature was 45°C, the detection wavelength was 210 nm, and the reference solution contained approximately 0.25 mg/mL erythromycin A diluted with pH 5.5 phosphate buffer.

#### 2.2.3 Statistics

The Mahalanobis distance between the dissolution curves was calculated using Excel 2007 (Microsoft, Redmond, WA, United States) and the DDSolver add-in (33). The Gompertz model was first used to parameterize the dissolution values at each point of the dissolution curves and then to calculate the Mahalanobis distance. The formula for the Gompertz model is as follows (Yi et al., 1997):

$$x\left(t\right)=x_{\max}\exp\left[-\alpha e^{-\beta \log\left(t\right)}\right]$$ (1)

where *x(t)* is the percent dissolution at time *t*, *x*_*max*_ is the maximum dissolution value, i.e., 100, *α* is the undissolved amount at *t* = 1, and *β* is the amount dissolved per unit time, i.e., the dissolution rate. The formula for calculating the Mahalanobis distance is as follows:

$$D_{M}=\sqrt{{\left(X_{T}-X_{R}\right)}^{\prime }S_{pooled}^{-1}\left(X_{T}-X_{R}\right)}$$ (2)

where $S_{pooled}=\left(S_{T}+S_{R}\right)/2$ is the variance-covariance matrix of the two sample batches, *X*_*T*_ and *X*_*R*_ are vectors of the mean values of the dissolution curves of the two sample batches, and *S*_*T*_ and *S*_*R*_ are the variance-covariance matrices of the dissolution curves of the two sample batches.

SPSS Statistics 23 (SPSS Inc., Chicago, IL, United States of America) was used for Pearson correlation analysis.

## 3 Results and discussions

### 3.1 Differences in the coating of enteric-coated erythromycin tablets produced by different processes determined using Raman mapping

**FIGURE 2:**
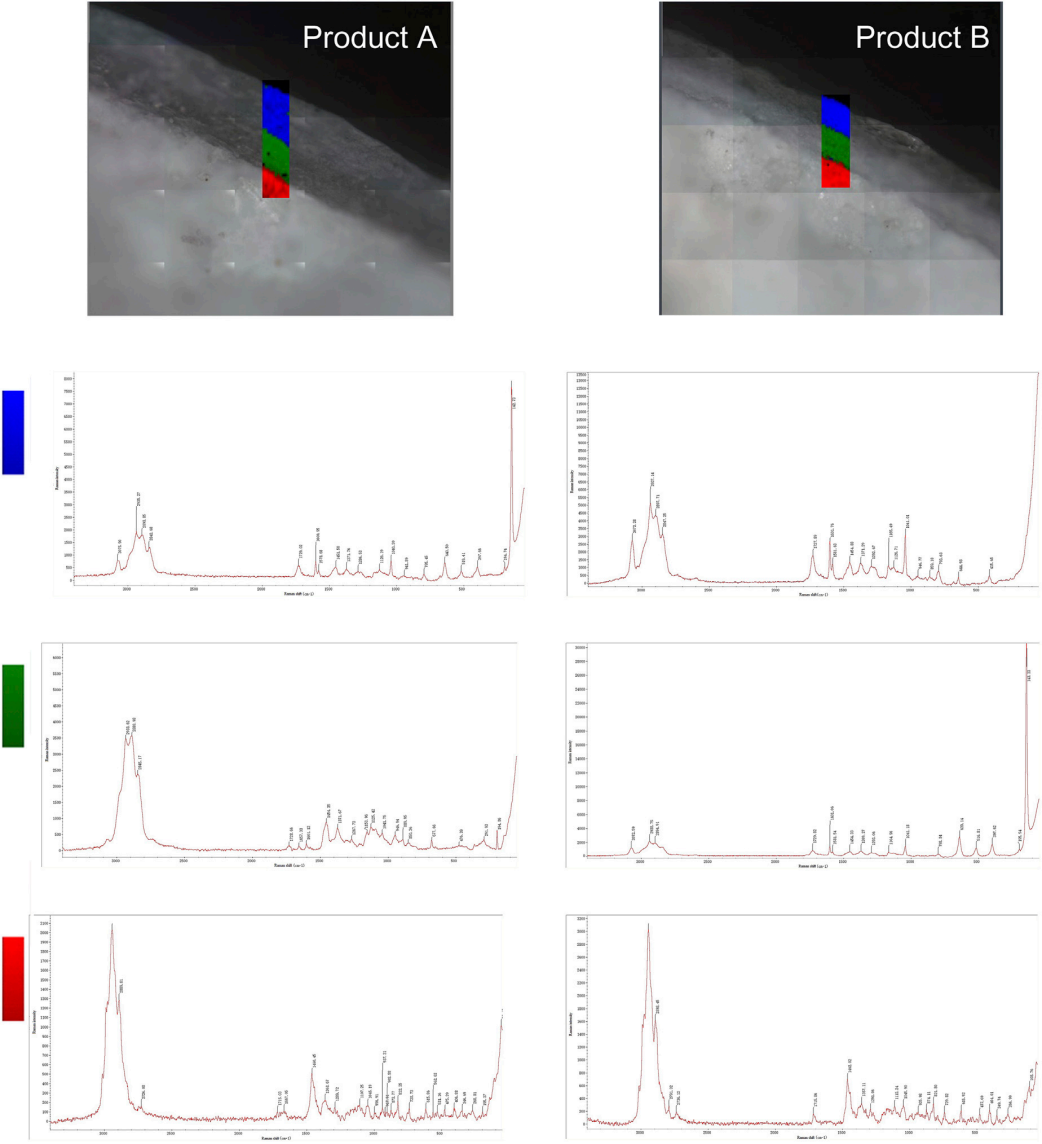
Raman mapping of the inner and outer coatings and tablet cores of the two product types. The rectangular area shows the imaging analysis of the outer coating in blue, the inner coating in green, and the tablet core in red.

The two types of enteric-coated erythromycin tablets were divided into two layers (Figure 2). Although it was difficult to determine whether the coating was layered from the color by observing its cross-section under an optical microscope, Raman mapping analysis showed that the coatings of both preparations were clearly divided into two layers. Further comparison of the Raman spectra of the same parts of the two formulations showed that the Raman spectra of the core part of the tablets were the same, suggesting that the API and excipients used were similar. However, the spectra of the two formulations had obvious differences between the outer and inner coatings. The spectra of the outer coating had small differences, which were only manifested in the differences in the peaks at Raman shifts of 142.73, 194.74, and 397.66 cm^−1^for product A and 415.60 cm^−1^ for product B. The differences in the inner layer of the coating were larger. The major difference was that product A had peaks at 291.93, 677.66, 850.36, 889.95, and 1,125.42 cm^−1^, whereas product B had major peaks at 397.62, 516.81, and 639.14 cm^−1^. This indicates that both formulations had different coating materials for the inner and outer coatings. These results suggest that Raman mapping is not only capable of distinguishing the coating from the tablet core based on spectral differences and determining the boundary between different coating layers but could also be used to determine the similarities and differences in coating formulations. Thus, the coating formulation process can be more accurately characterized, and the thickness of the coating can be measured.

### 3.2 Evaluation of the coating process characteristics of different enteric-coated erythromycin tablets

The coating thicknesses of the six sides of products A and B were determined separately (Table 1) and used to characterize the two formulation processes.

**TABLE 1:** Coating thickness (μm) of the six sides of each sample batch.

Samples		F1		F2		S3		S4		S5		S6	
		Outer	Inner	Outer	Inner	Outer	Inner	Outer	Inner	Outer	Inner	Outer	Inner
A-1	1	49.9 ± 3.4	22.4 ± 4.2	37.3 ± 8.2	26.9 ± 5.8	49.7 ± 1.7	24.5 ± 2.2	45.7 ± 4.3	28.1 ± 4.1	56.5 ± 7.8	33.3 ± 6.0	54.8 ± 8.2	35.5 ± 12.4
	2	65.5 ± 8.2	20.9 ± 7.4	63.9 ± 7.3	27.9 ± 4.2	62.7 ± 3.1	31.7 ± 5.9	57.6 ± 4.6	21.8 ± 3.2	77.6 ± 4.0	31.3 ± 1.1	71.3 ± 6.6	29.8 ± 4.5
	3	54.3 ± 5.2	20.1 ± 3.7	48.3 ± 10.2	25.5 ± 11.3	46.3 ± 3.0	19.1 ± 2.7	56.5 ± 6.5	18.9 ± 3.0	56.0 ± 5.5	24.2 ± 3.8	62.2 ± 11.1	22.6 ± 2.9
	4	62.8 ± 6.8	20.1 ± 5.9	57.2 ± 4.1	24.0 ± 8.2	58.0 ± 3.3	25.4 ± 3.6	53.5 ± 8.4	22.5 ± 2.4	70.0 ± 6.0	33.5 ± 5.9	62.5 ± 6.6	24.0 ± 2.8
	5	66.7 ± 13.9	22.7 ± 2.4	62.7 ± 9.1	29.3 ± 3.9	58.4 ± 2.4	25.0 ± 5.6	60.2 ± 2.7	23.2 ± 5.4	70.7 ± 3.5	25.9 ± 6.2	78.4 ± 11.0	44.2 ± 10.2
	6	41.8 ± 4.8	19.6 ± 5.6	45.8 ± 1.5	21.6 ± 8.6	33.5 ± 7.6	14.3 ± 4.8	45.6 ± 1.1	21.4 ± 7.8	59.8 ± 7.0	19.4 ± 2.8	53.4 ± 11.6	22.6 ± 5.7
Mean		56.8 ± 9.9	21.0 ± 1.3	52.5 ± 10.5	25.9 ± 2.8	51.4 ± 10.7	23.3 ± 5.9	53.2 ± 6.2	22.6 ± 3.0	65.1 ± 8.9	27.9 ± 5.7	63.8 ± 9.6	29.8 ± 8.7
A-2	1	44.9 ± 5.7	29.2 ± 7.2	55.0 ± 2.1	26.1 ± 7.0	43.1 ± 0.5	28.4 ± 3.2	45.2 ± 8.1	24.4 ± 3.7	72.5 ± 7.7	42.6 ± 9.6	65.3 ± 7.2	33.3 ± 4.8
	2	44.6 ± 8.7	29.8 ± 2.6	49.0 ± 6.7	23.6 ± 3.4	44.6 ± 1.6	25.4 ± 4.8	51.7 ± 5.9	23.3 ± 4.6	75.6 ± 5.6	23.0 ± 2.3	68.6 ± 9.8	28.9 ± 5.0
	3	68.5 ± 7.3	29.6 ± 7.7	48.6 ± 3.8	29.3 ± 5.8	50.7 ± 8.8	29.0 ± 6.8	49.3 ± 5.9	29.5 ± 1.5	67.5 ± 6.2	31.4 ± 3.4	83.3 ± 9.6	36.7 ± 3.9
	4	68.0 ± 3.9	19.6 ± 4.8	58.4 ± 9.2	27.8 ± 5.0	68.1 ± 2.3	18.3 ± 6.0	64.7 ± 3.5	23.2 ± 3.4	64.2 ± 4.1	26.0 ± 6.1	75.5 ± 8.7	25.6 ± 5.8
	5	53.3 ± 6.9	28.7 ± 6.2	56.0 ± 2.2	32.2 ± 2.5	54.0 ± 6.1	30.0 ± 6.6	50.1 ± 7.6	31.8 ± 3.4	65.6 ± 8.6	35.8 ± 9.7	73.4 ± 6.6	28.5 ± 6.9
	6	53.8 ± 4.4	25.3 ± 1.7	49.1 ± 8.6	21.8 ± 2.7	50.3 ± 8.7	19.9 ± 5.2	52.3 ± 6.7	19.7 ± 3.1	65.4 ± 3.5	30.5 ± 5.4	67.4 ± 5.4	27.5 ± 4.6
Mean		55.5 ± 10.6	27.0 ± 4.0	52.7 ± 4.3	26.8 ± 3.8	51.8 ± 9.0	25.2 ± 5.0	52.2 ± 6.6	25.3 ± 4.5	68.5 ± 4.6	31.5 ± 7.0	72.3 ± 6.6	30.1 ± 4.1
A-3	1	63.0 ± 6.1	29.2 ± 4.8	66.0 ± 2.5	22.7 ± 3.2	58.0 ± 7.1	23.1 ± 3.3	67.6 ± 7.1	25.4 ± 6.9	60.9 ± 6.4	39.8 ± 9.5	77.9 ± 5.4	37.8 ± 5.7
	2	46.2 ± 3.7	24.6 ± 7.2	57.8 ± 2.2	30.9 ± 5.2	56.1 ± 7.3	23.5 ± 4.1	49.8 ± 5.7	19.9 ± 3.1	49.5 ± 6.5	27.4 ± 1.8	60.9 ± 3.1	32.8 ± 6.8
	3	70.1 ± 5.8	26.2 ± 6.4	61.7 ± 1.4	29.9 ± 6.1	54.5 ± 17.2	24.2 ± 1.3	59.6 ± 5.8	22.4 ± 3.6	56.5 ± 16.0	27.9 ± 3.2	60.7 ± 18.2	28.6 ± 6.8
	4	60.6 ± 4.5	26.8 ± 3.4	61.3 ± 3.9	26.3 ± 2.6	47.9 ± 6.9	23.3 ± 3.7	60.8 ± 5.8	25.9 ± 8.3	62.5 ± 14.3	24.8 ± 4.5	62.2 ± 8.9	23.5 ± 1.5
	5	64.4 ± 5.8	29.6 ± 2.1	69.0 ± 2.7	28.6 ± 4.9	64.3 ± 6.8	31.0 ± 4.8	57.9 ± 5.4	34.3 ± 5.5	71.4 ± 6.1	33.4 ± 7.5	69.2 ± 9.4	33.6 ± 8.5
	6	54.8 ± 7.5	25.1 ± 3.0	58.2 ± 2.3	24.2 ± 1.8	51.6 ± 3.8	25.1 ± 8.6	53.2 ± 3.9	25.3 ± 4.9	71.2 ± 14.7	28.3 ± 6.8	66.5 ± 11.6	26.9 ± 2.4
Mean		59.9 ± 8.3	26.9 ± 2.1	62.3 ± 4.4	27.1 ± 3.3	55.4 ± 5.6	25.0 ± 3.0	58.1 ± 6.2	25.5 ± 4.9	62.0 ± 8.5	30.3 ± 5.5	66.2 ± 6.6	30.5 ± 5.2
A-5	1	49.7 ± 6.8	48.6 ± 5.4	50.3 ± 6.1	42.6 ± 5.1	49.4 ± 6.0	42.0 ± 2.2	52.1 ± 6.5	37.4 ± 6.6	55.9 ± 5.5	39.8 ± 4.1	54.4 ± 9.4	52.3 ± 6.0
	2	63.3 ± 9.5	46.5 ± 3.5	67.9 ± 9.4	43.1 ± 3.8	60.5 ± 2.8	39.0 ± 2.6	59.4 ± 5.8	37.3 ± 5.7	67.3 ± 8.3	37.9 ± 6.9	65.8 ± 3.6	44.9 ± 7.3
	3	64.4 ± 5.0	38.1 ± 1.4	59.9 ± 4.2	40.7 ± 1.9	62.7 ± 5.6	33.0 ± 5.4	59.3 ± 6.0	33.3 ± 3.6	62.2 ± 4.8	38.0 ± 3.1	68.2 ± 4.4	39.3 ± 4.7
	4	59.4 ± 8.3	38.9 ± 4.2	57.4 ± 5.0	48.5 ± 2.6	64.4 ± 5.6	40.5 ± 5.2	56.3 ± 10.0	38.6 ± 5.3	74.0 ± 7.3	42.5 ± 4.9	52.2 ± 3.3	50.0 ± 5.5
	5	62.6 ± 6.4	41.1 ± 4.7	55.9 ± 10.5	42.7 ± 6.4	53.3 ± 4.5	44.2 ± 5.0	55.1 ± 6.2	42.1 ± 2.9	52.9 ± 6.1	47.2 ± 4.1	55.6 ± 5.8	53.2 ± 5.4
	6	84.5 ± 3.8	42.5 ± 5.2	65.3 ± 4.9	34.4 ± 4.4	64.5 ± 7.1	33.2 ± 3.6	57.0 ± 9.9	31.3 ± 4.3	64.4 ± 7.5	42.1 ± 3.4	58.7 ± 5.7	29.7 ± 6.1
Mean		64.0 ± 11.4	42.6 ± 4.2	59.4 ± 6.4	42.0 ± 4.6	59.1 ± 6.3	38.6 ± 4.6	56.5 ± 2.8	36.7 ± 3.9	62.8 ± 7.7	41.2 ± 3.5	59.2 ± 6.5	44.9 ± 9.1
A-6	1	58.8 ± 6.8	35.5 ± 4.1	53.8 ± 5.3	40.0 ± 4.4	56.1 ± 4.8	37.8 ± 5.7	44.3 ± 4.5	31.9 ± 3.9	56.8 ± 4.5	39.8 ± 4.9	61.5 ± 6.3	41.7 ± 4.3
	2	81.7 ± 11.9	42.0 ± 3.4	54.4 ± 5.9	44.2 ± 7.9	46.6 ± 4.4	40.4 ± 4.1	59.9 ± 4.0	32.9 ± 4.9	50.9 ± 8.1	46.8 ± 2.7	78.3 ± 3.2	39.0 ± 4.8
	3	64.1 ± 5.9	41.7 ± 6.4	63.6 ± 5.4	38.8 ± 5.1	54.9 ± 10.5	42.6 ± 5.3	58.2 ± 5.5	39.1 ± 3.0	62.2 ± 6.1	49.7 ± 7.7	50.1 ± 6.1	37.9 ± 6.8
	4	74.0 ± 7.5	38.3 ± 4.8	65.9 ± 11.0	39.9 ± 5.5	51.1 ± 7.7	38.7 ± 6.7	67.3 ± 10.6	30.0 ± 7.0	60.9 ± 6.1	38.8 ± 5.2	60.7 ± 4.6	41.9 ± 4.9
	5	59.1 ± 12.7	49.1 ± 9.2	60.4 ± 10.9	41.3 ± 11.3	46.9 ± 3.7	38.1 ± 1.8	52.8 ± 3.7	35.1 ± 2.7	52.1 ± 3.4	46.8 ± 2.8	52.0 ± 6.5	46.8 ± 11.2
	6	78.0 ± 8.4	48.1 ± 2.2	74.7 ± 13.6	45.8 ± 4.2	62.6 ± 3.7	37.3 ± 10.8	67.7 ± 3.7	44.6 ± 2.7	78.9 ± 3.6	48.8 ± 4.4	74.6 ± 9.1	53.2 ± 3.1
Mean		69.3 ± 9.9	42.5 ± 5.3	62.1 ± 7.8	41.7 ± 2.8	53.1 ± 6.1	39.1 ± 2.0	58.4 ± 8.9	35.6 ± 5.4	60.3 ± 10.2	45.1 ± 4.7	62.9 ± 11.5	43.4 ± 5.7
A-7	1	80.2 ± 6.6	35.7 ± 5.8	70.6 ± 5.6	37.8 ± 3.0	88.1 ± 8.4	43.5 ± 9.7	60.6 ± 6.4	39.3 ± 5.9	73.1 ± 11.1	37.4 ± 2.0	73.9 ± 9.9	35.5 ± 4.2
	2	67.7 ± 7.4	49.6 ± 3.3	59.6 ± 7.1	34.0 ± 2.1	57.7 ± 8.6	35.8 ± 2.2	57.1 ± 5.6	36.7 ± 4.2	58.8 ± 11.6	42.2 ± 7.4	63.3 ± 7.8	46.5 ± 4.9
	3	66.4 ± 6.3	42.2 ± 6.6	50.1 ± 3.5	36.8 ± 2.6	67.8 ± 4.0	36.6 ± 1.3	59.2 ± 5.9	31.4 ± 5.5	60.3 ± 3.5	45.2 ± 3.7	54.3 ± 4.2	42.5 ± 3.9
	4	78.8 ± 7.7	37.4 ± 2.3	70.9 ± 2.5	37.4 ± 4.1	66.6 ± 8.6	34.6 ± 6.6	66.7 ± 10.6	31.9 ± 6.7	61.2 ± 12.3	38.3 ± 5.1	69.7 ± 4.4	40.2 ± 4.4
	5	74.4 ± 10.1	37.6 ± 1.5	75.4 ± 5.7	48.8 ± 5.5	60.5 ± 8.8	43.1 ± 5.0	61.4 ± 6.1	34.6 ± 6.2	72.3 ± 9.4	43.0 ± 5.5	59.3 ± 3.9	41.3 ± 3.9
	6	62.0 ± 3.2	37.9 ± 3.2	51.5 ± 5.8	35.3 ± 5.4	56.7 ± 5.6	30.7 ± 2.8	56.1 ± 5.8	34.0 ± 6.7	61.0 ± 4.4	38.4 ± 1.6	69.8 ± 13.9	35.9 ± 4.5
Mean		71.6 ± 7.3	40.1 ± 5.1	63.0 ± 10.8	38.3 ± 5.3	66.2 ± 11.7	37.4 ± 5.0	60.2 ± 3.8	34.6 ± 3.0	64.4 ± 6.4	40.7 ± 3.1	65.1 ± 7.4	40.3 ± 4.2
B-1	1	39.2 ± 2.5	36.6 ± 1.8	37.6 ± 4.2	32.1 ± 5.0	36.4 ± 1.7	31.5 ± 2.1	31.4 ± 1.3	28.5 ± 3.1	41.5 ± 6.9	30.6 ± 2.5	39.7 ± 4.0	27.4 ± 2.2
	2	38.7 ± 6.4	36.1 ± 5.0	32.9 ± 2.2	41.1 ± 9.3	26.6 ± 4.7	33.5 ± 7.1	35.7 ± 3.6	29.3 ± 4.1	33.6 ± 1.9	36.0 ± 4.3	30.4 ± 6.4	31.9 ± 6.9
	3	43.9 ± 6.0	34.8 ± 3.8	42.1 ± 3.3	37.7 ± 5.5	42.6 ± 4.8	27.0 ± 3.2	42.1 ± 6.3	27.7 ± 3.1	40.8 ± 6.9	32.5 ± 2.2	39.7 ± 1.9	32.1 ± 3.0
	4	35.1 ± 4.2	34.3 ± 3.0	39.6 ± 2.3	35.3 ± 2.3	27.6 ± 3.4	28.4 ± 3.7	46.2 ± 1.4	30.2 ± 3.8	33.8 ± 4.1	34.7 ± 2.1	33.1 ± 6.3	33.9 ± 2.6
	5	35.8 ± 1.4	35.7 ± 4.3	36.8 ± 4.9	28.9 ± 2.9	32.2 ± 2.6	27.8 ± 2.7	35.5 ± 1.9	28.2 ± 3.3	29.1 ± 4.0	40.7 ± 7.4	29.9 ± 3.0	37.9 ± 5.0
	6	41.2 ± 5.5	38.8 ± 6.1	35.7 ± 8.2	34.2 ± 6.0	32.9 ± 1.8	33.7 ± 3.8	45.4 ± 5.2	36.8 ± 5.9	43.0 ± 3.9	39.6 ± 7.8	33.7 ± 5.8	41.5 ± 4.3
Mean		39.0 ± 3.3	36.1 ± 1.6	37.4 ± 3.2	34.9 ± 4.3	33.1 ± 5.9	30.3 ± 3.0	39.4 ± 6.0	30.1 ± 3.4	37.0 ± 5.6	35.7 ± 3.9	34.4 ± 4.4	34.1 ± 5.0
B-2	1	32.1 ± 1.8	35.0 ± 0.6	33.8 ± 4.1	34.5 ± 1.6	27.1 ± 3.7	31.2 ± 2.6	25.4 ± 3.5	31.1 ± 2.6	35.2 ± 6.7	33.5 ± 1.9	28.8 ± 4.0	33.8 ± 2.3
	2	32.3 ± 4.4	33.1 ± 5.9	25.1 ± 5.2	35.6 ± 5.1	25.8 ± 7.3	29.2 ± 2.9	21.4 ± 2.9	30.7 ± 3.9	25.9 ± 4.3	26.0 ± 2.9	25.9 ± 2.4	36.3 ± 4.6
	3	25.9 ± 3.2	28.0 ± 1.6	23.7 ± 2.3	27.2 ± 4.6	24.1 ± 3.4	25.7 ± 3.3	21.8 ± 3.1	27.0 ± 2.1	24.2 ± 4.6	31.1 ± 2.7	28.6 ± 4.6	26.4 ± 2.5
	4	30.1 ± 4.0	32.9 ± 2.2	28.6 ± 3.4	29.0 ± 4.5	30.2 ± 4.9	29.4 ± 1.9	20.9 ± 4.2	26.3 ± 2.8	33.9 ± 4.1	31.0 ± 4.2	25.3 ± 4.5	31.2 ± 5.2
	5	40.0 ± 5.7	38.5 ± 3.2	47.5 ± 3.7	37.1 ± 1.9	44.0 ± 4.4	34.9 ± 4.7	47.2 ± 5.1	28.1 ± 3.1	39.4 ± 2.2	36.0 ± 2.7	53.0 ± 6.4	36.8 ± 4.9
	6	27.8 ± 4.3	39.0 ± 4.9	28.3 ± 5.0	32.2 ± 2.3	24.5 ± 5.6	31.9 ± 3.4	24.2 ± 3.0	30.0 ± 4.2	31.1 ± 5.0	28.3 ± 3.9	28.6 ± 6.8	35.2 ± 4.2
Mean		31.4 ± 4.9	34.4 ± 4.1	31.2 ± 8.7	32.6 ± 3.8	29.3 ± 7.5	30.4 ± 3.1	26.8 ± 10.1	28.9 ± 2.0	31.6 ± 5.8	31.0 ± 3.6	31.7 ± 10.6	33.3 ± 3.9
B-3	1	34.3 ± 5.5	37.0 ± 3.5	32.8 ± 5.7	34.3 ± 5.7	30.4 ± 4.3	30.5 ± 1.0	32.5 ± 3.2	31.3 ± 2.8	25.3 ± 2.3	29.8 ± 3.9	34.7 ± 3.4	34.0 ± 5.0
	2	38.9 ± 4.3	34.8 ± 1.6	34.1 ± 2.7	34.1 ± 5.4	30.7 ± 2.2	31.1 ± 3.5	30.7 ± 2.6	27.4 ± 2.5	31.7 ± 3.7	36.8 ± 5.0	31.9 ± 2.7	29.9 ± 2.9
	3	40.2 ± 7.1	26.8 ± 4.2	41.2 ± 3.3	27.8 ± 2.3	41.1 ± 4.4	22.9 ± 5.4	33.0 ± 5.7	25.4 ± 5.4	37.0 ± 5.8	22.9 ± 2.9	34.0 ± 5.2	28.2 ± 3.1
	4	34.7 ± 7.0	35.6 ± 4.2	27.8 ± 4.2	33.9 ± 5.3	28.4 ± 5.7	31.4 ± 3.6	27.8 ± 4.3	33.8 ± 5.4	29.7 ± 6.3	34.0 ± 2.9	38.3 ± 5.7	35.0 ± 3.6
	5	36.7 ± 4.5	38.3 ± 2.1	29.9 ± 4.6	33.2 ± 4.3	31.9 ± 2.0	33.8 ± 3.0	31.6 ± 2.5	31.7 ± 1.6	34.2 ± 6.0	36.6 ± 3.1	34.2 ± 4.6	36.0 ± 3.3
	6	36.9 ± 8.3	35.1 ± 2.8	25.2 ± 3.8	27.5 ± 2.5	29.1 ± 4.0	24.8 ± 3.3	33.7 ± 2.6	29.0 ± 3.1	30.5 ± 4.5	35.5 ± 7.1	25.7 ± 1.8	34.2 ± 4.0
Mean		36.9 ± 2.3	34.6 ± 4.0	31.8 ± 5.6	31.8 ± 3.2	31.9 ± 4.6	29.1 ± 4.2	31.6 ± 2.1	29.8 ± 3.1	31.4 ± 4.0	32.6 ± 5.4	33.1 ± 4.2	32.9 ± 3.1
B-4	1	32.6 ± 3.7	33.3 ± 3.6	32.4 ± 2.9	36.3 ± 2.4	28.3 ± 4.2	34.5 ± 2.2	32.6 ± 5.3	28.1 ± 4.6	27.4 ± 5.6	29.1 ± 3.7	35.2 ± 3.0	39.2 ± 5.0
	2	33.2 ± 3.0	37.3 ± 3.1	47.6 ± 8.4	39.4 ± 2.6	30.0 ± 4.8	33.4 ± 3.5	44.9 ± 4.2	32.4 ± 1.6	35.0 ± 1.6	37.3 ± 3.2	39.4 ± 3.4	38.8 ± 3.3
	3	28.7 ± 6.5	36.6 ± 2.7	34.7 ± 4.5	38.5 ± 3.8	28.6 ± 2.3	29.6 ± 2.0	26.6 ± 3.6	37.3 ± 7.6	31.2 ± 2.9	39.3 ± 8.0	28.2 ± 4.8	37.6 ± 3.9
	4	42.0 ± 3.8	32.4 ± 4.3	34.0 ± 1.1	41.5 ± 4.2	29.8 ± 4.8	30.2 ± 2.0	34.2 ± 4.2	31.1 ± 2.7	39.9 ± 5.0	33.8 ± 7.2	31.5 ± 2.8	36.9 ± 3.9
	5	43.9 ± 4.8	31.4 ± 4.1	51.9 ± 2.7	33.6 ± 2.4	42.4 ± 5.9	30.6 ± 3.7	43.5 ± 3.1	30.6 ± 4.3	38.6 ± 4.6	30.6 ± 3.4	47.3 ± 5.7	37.9 ± 4.3
	6	30.1 ± 3.9	41.1 ± 8.9	31.3 ± 4.5	36.7 ± 3.5	26.5 ± 4.4	35.7 ± 4.6	30.3 ± 1.7	35.5 ± 4.6	31.5 ± 5.3	36.6 ± 3.9	32.2 ± 3.5	34.2 ± 2.1
Mean		35.1 ± 6.3	35.4 ± 3.7	38.6 ± 8.8	37.6 ± 2.7	30.9 ± 5.7	32.3 ± 2.5	35.4 ± 7.3	32.5 ± 3.4	34.0 ± 4.8	34.4 ± 4.0	35.6 ± 6.9	37.4 ± 1.8

The coating thicknesses of the six sides of product A were similar, with thick outer and thin inner coatings. For product A, the outer coating thickness [mean ± standard deviation (SD)] was 60.5 ± 16.4 µm. The inner coating thicknesses of the products with high and low strengths differed; the inner coating thickness of A-1, A-2, and A-3 was 26.8 ± 4.4 µm, while that of A-5, A-6, and A-7 was 42.9 ± 6.3 µm. However, the outer coating thicknesses of the different sides were clearly uneven, with the largest difference in F1, which had an SD of 9.6 µm.

The outer and inner layer thicknesses of product B were similar, with the thickness of the outer layer being 33.7 ± 5.8 μm and that of the inner layer being 34.4 ± 3.4 μm. There were no significant differences in the thicknesses of the coatings on each side.

Taken together, the coating differences between the two types of enteric-coated erythromycin tablets were primarily in the outer coating. The outer coating thickness of product A was significantly larger than that of product B, and the coating thickness of product B was more uniform among the six sides of each tablet, suggesting that the coating process is not identical for the two formulations.

### 3.3 Disintegration-release characteristics of enteric-coated erythromycin tablets

Usually, the dissolution curve of enteric-coated tablets is measured using pH 6.8 phosphate buffer as the dissolution medium. At this pH, the coating on each side of the enteric-coated tablets dissolves and cracks rapidly, releasing the drug. However, the dissolution profile is not sufficiently differentiated, making identifying the differences in the formulation processes of different manufacturers difficult (Supplementary Material S3). Therefore, selecting a dissolution medium with an appropriate pH is necessary to allow enteric-coated tablets to dissolve slowly. Thus, a pH 5.5 phosphate buffer solution was selected as the dissolution medium. In this medium, enteric-coated erythromycin tablets began to dissolve gradually only after 15–30 min.

The two types of enteric-coated erythromycin tablets had different outer coating materials. The thickness and homogeneity of the outer coating were also different, and dissolution tests further compared their dissolution and release characteristics.

**FIGURE 3:**
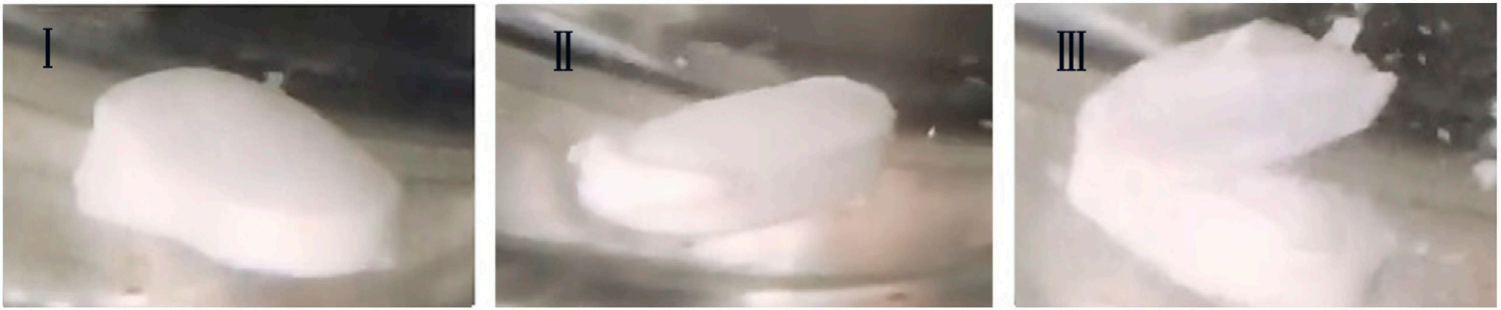
The three phases of the coating crack during dissolution process.

Real-time monitoring of dissolution showed that the coating dissolution crack process was similar for both formulations and could be divided into three phases (Figure 3) (Supplementary Materials S1, S2). In phase I, the side coating begins to dissolve; in phase II, the side coating cracks; in phase III, the side coating almost completely breaks open, and the face coating detaches from the core. Product A dissolved slowly, taking 23–33 min to phase I from the beginning of dissolution, and it took a long time for the side coating to crack after it started to dissolve. The interval between phases I and III was approximately 8 min. Product B dissolved more rapidly, starting to dissolve within 20 min (entering phase I) and cracking rapidly after the coating dissolved. The interval between phases I and III was approximately 5 min.

For dissolution profile determination, the start of phase I was defined as time 0. After entering phase III, the tablet core was exposed to the dissolution medium over a large area and began to dissolve rapidly, releasing the API. Due to the protective effect of coating on the tablet core, the core only begins to dissolve when the coating dissolves and cracks. At this stage, the formulation and thickness of the coating are key factors affecting the dissolution rate. When the coating is completely removed from the core, the dissolution rate mainly depends on the characteristics of the core, such as the particle size of the granules and hardness of the tablet. This demonstrated that coating formulation and processing affect the disintegration time of enteric-coated erythromycin tablets and their dissolution behavior.

### 3.4 Dissolution characteristics of different products

**FIGURE 4:**
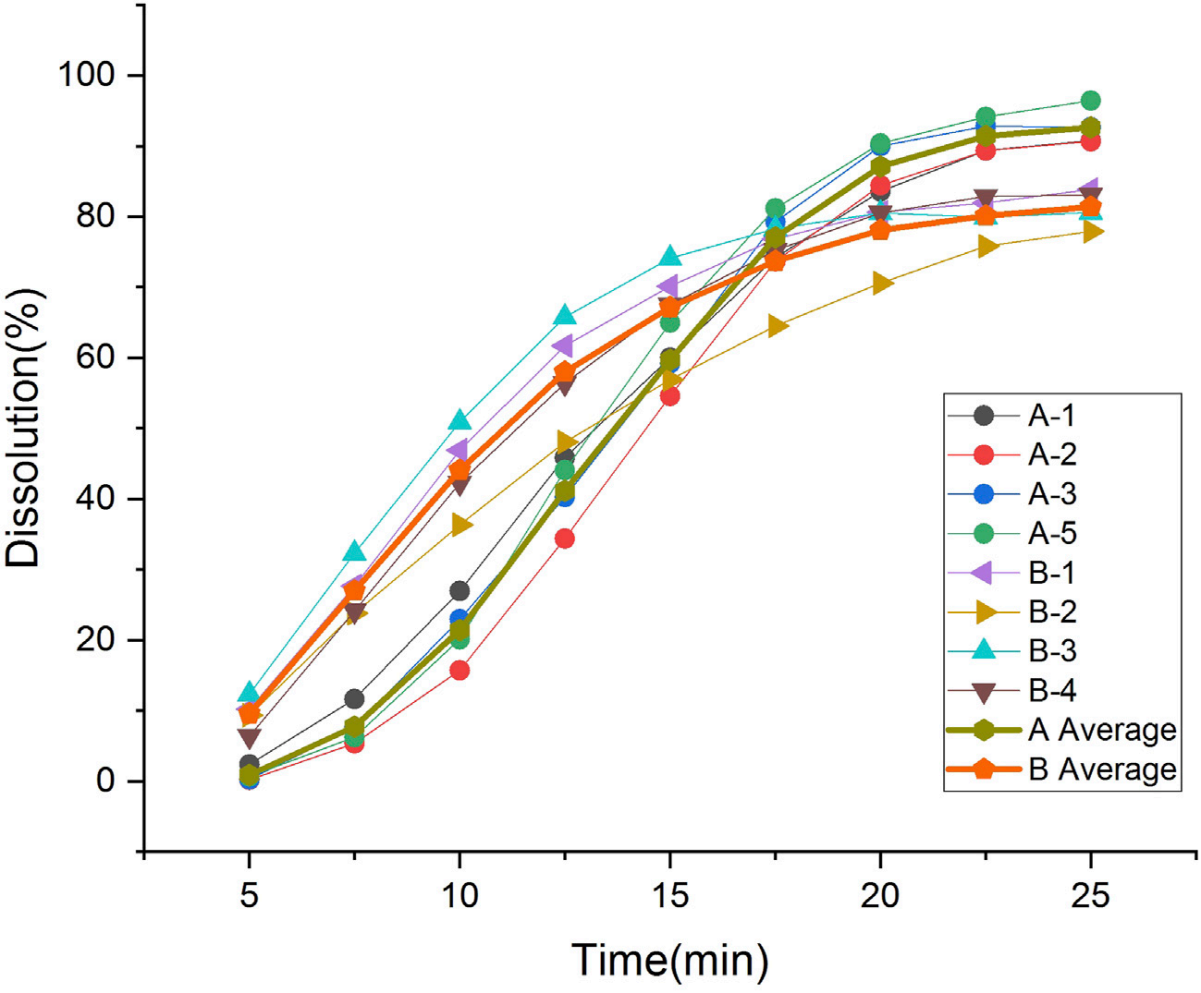
Dissolution and average profiles of four batches for each products.

The two types of enteric-coated erythromycin tablets with different coating formulations and processes exhibited different dissolution characteristics in the dissolution test, producing different dissolution profile (Figure 4). The dissolution of product A was initially slow, and the amount dissolved was clearly lower than that of product B. The degree of dissolution was less than 3% at 5 min, increased after 7.5 min, was over 80% at 20 min, and over 90% at the end of dissolution. It took nearly 8 min to transition from phase I to phase III, and after entering Phase III, the core dissolution rate accelerated, manifested as a fast dissolution rate within 10–20 min. The *inter*-tablet variability in dissolution amounts indicated the differences in the entry into phase III between tablets. The dissolution rate was most rapid and largest in difference between tablets at 10–20 min. Product B coating cracked early, with a rapid dissolution onset. The level of dissolution reached 6%–12% at 5 min, was over 35% at 10 min, and 80% at 20 min. There was a large difference in the amount of tablets dissolved in the first 15 min, but the difference gradually decreased thereafter. The *inter*-tablet variability of dissolution of both preparations was large in the fast dissolution interval, due to the differences in the time of entry into phase III between the tablets. These differences gradually decreased when the dissolution rate leveled off (Table 2). Thus, the rapid dissolution rate is the main reason for the large *inter*-tablet variability in dissolution amounts, which is caused by the formulation and thickness of the coating. The coating materials of the two types of products and their tolerance to the dissolution medium differ. The outer coating of product B is relatively thin, and it dissolved and cracked quickly, the differences in the time of entry into phase III is significant, and the variation in the dissolution amounts during the initial stage (rapid dissolution stage) of the dissolution was also greater.

**TABLE 2:** Standard deviation of dissolution values at each time point for each batch of the six tablet samples.

Samples	5 min (%)	7.5 min (%)	10 min (%)	12.5 min (%)	15 min (%)	17.5 min (%)	20 min (%)	22.5 min (%)	25 min (%)
A-1	2.4	12.7	18.4	20.0	19.0	13.4	7.4	1.7	2.8
A-2	0.2	6.3	10.1	10.2	15.2	8.8	6.4	3.9	3.1
A-3	0.3	5.1	6.1	9.9	10.6	5.1	2.4	0.9	1.2
A-5	0.9	2.3	4.8	8.6	11.5	12.7	12.4	10.0	4.8
B-1	10.0	9.4	6.6	4.6	3.3	2.1	0.9	1.4	5.5
B-2	9.1	17.4	22.1	23.4	23.0	20.7	15.9	8.1	5.3
B-3	13.8	18.3	18.0	14.6	12.0	8.5	5.2	5.0	2.6
B-4	8.5	13.2	11.9	9.1	5.8	3.8	2.8	2.0	2.1

The Mahalanobis distance of the dissolution profile of each sample batch was calculated as previously described (Zhang et al., 2010) (Supplementary Material S3). The Mahalanobis distance between the dissolution curves of each batch of company A products was below 2.5, similar to that of each batch of company B products. The Mahalanobis distance between the dissolution curves of each batch of products from the two companies was over 2.5, suggesting that differences in enteric-coated erythromycin tablet coating formulations and processing were the principal reason for dissolution profile differences.

### 3.5 Correlation analysis between coating thickness and dissolution profiles

Both coating thickness and dissolution profiles reflect the differences between the processing of the two products. Coating thickness and uniformity in different parts may correlate with dissolution behavior. Pearson correlation analyses were performed on the outer coating thicknesses of different sides of products A and B, as well as the SD of the thicknesses with the amount of dissolution and the SD of the respective dissolution curves for the nine time points. Due to the symmetry of F1 and F2, S3 and S4, S5 and S6 in the tablets, it is reasonable to use the mean value of symmetry plane thickness and SD. Therefore, Pearson correlation analysis was performed using the mean values of F1 and F2, S3 and S4, S5 and S6. The results showed that the average thickness of S5 and S6 of product A was strongly negatively correlated with the dissolution at 15 min (Table 3). The coatings of both types of products started to dissolve and crack from S5 or S6 at the time of dissolution. Due to the thicker coatings of S5 and S6 of product A, the coating dissolved slowly and the dissolution rate at 15 min was low. The coating thickness at this site directly affects the amount of dissolution, suggesting that the outer layer thickness of side S5 and S6 is critical in the coating process for product A.

**TABLE 3:** Results of Pearson correlation analysis for product A and product B.

Average of S5 and S6 (outer)		SD of dissolution at 7.5 min	SD of dissolution at 10 min	Dissolution at 15 min
Product A	Pearson Correlation	—	—	−0.965^a^
	Sig. (2-tailed)	—	—	0.035
	N	—	—	4
Product B	Pearson Correlation	−0.957^a^	−0.983^a^	—
	Sig. (2-tailed)	0.043	0.017	—
	N	4	4	—

^a^ Correlation is significant at the 0.05 (2-tailed).

The outer coating of product B was thinner and more uniform in thickness than that of product A. There was no significant correlation between the variation in coating thickness and the dissolution amount SDs, but the outer coating average thickness of S5 and S6 was strongly negatively correlated with the SD of the dissolution amount at 7.5 and 10 min (Table 3). Particularly, the smaller thickness of the two side coatings was strongly correlated with the larger difference in dissolution amounts at these two time points, suggesting that the outer coating thickness of side S5 and S6 is also critical to the coating process for product B. When the outer coating thickness of side S5 and S6 was large, it caused low dissolution. When the coatings of these two sides are thin, they affect the *inter*-tablet variability of dissolution. This is in agreement with Ariyasu *et al.* (Korasa et al., 2016), who concluded that the side coating of enteric-coated tablets is critical following studies on the coating thickness of enteric-coated aspirin tablets.

## 4 Conclusion

Quality controls of enteric-coated tablets should emphasize the formulation process in addition to the quality of the API. The coating process is an important part of the formulation of enteric-coated tablets that may affect the quality of the formulation, which in turn affects *in vivo* release and efficacy. Analysis of the disintegration-release characteristics of the two enteric-coated erythromycin tablets revealed that the dissolution and cracking of the coating started from the side of the tablet, and the outer coating determined the dissolution rate. Although there are differences in the coating processes for the two types of enteric-coated erythromycin tablets, the thickness of the outer coating on the side is a critical quality attribute in both processes. The outer coating of product A is relatively thick, and the thickness of the outer coating on the side affects the dissolution amount. The outer coating of product B is relatively thin, resulting in a short cracking time and large variation and a significant difference in the initial dissolution amounts between tablets. In addition, the formulation and process of the tablet core affect the dissolution rate and final dissolution amount after phase III.

Enteric-coated erythromycin tablets contain two layers of coating. Raman mapping based on the spectral information of the inner and outer coating and the core of the tablets could clearly differentiate the boundary between the layers. Thus, the thickness of the two coating layers could be accurately determined, enabling the evaluation of coating process consistency. The coating thickness and uniformity of enteric-coated erythromycin tablets are important indicators reflecting control of the coating process. Coating uniformity is influenced by the equipment geometry, the coating solution properties, and a multitude of process parameters during coating (Wahl et al., 2019). In conclusion, the coating process of enteric-coated tablets can be evaluated rapidly and intuitively using Raman mapping.

## Data Availability

The original contributions presented in the study are included in the article/Supplementary Material, further inquiries can be directed to the corresponding authors.
