# Supplementary material for: Characterization of enteric-coated erythromycin tablets by Raman mapping and its pharmaceutical evaluation
Source: Front Chem. 2023 Oct 18;11:1270737. doi: 10.3389/fchem.2023.1270737 (PMC10619665; doi:10.3389/fchem.2023.1270737)
Supplement: Supplementary file 3 [file DataSheet1.docx]

Supplementary material


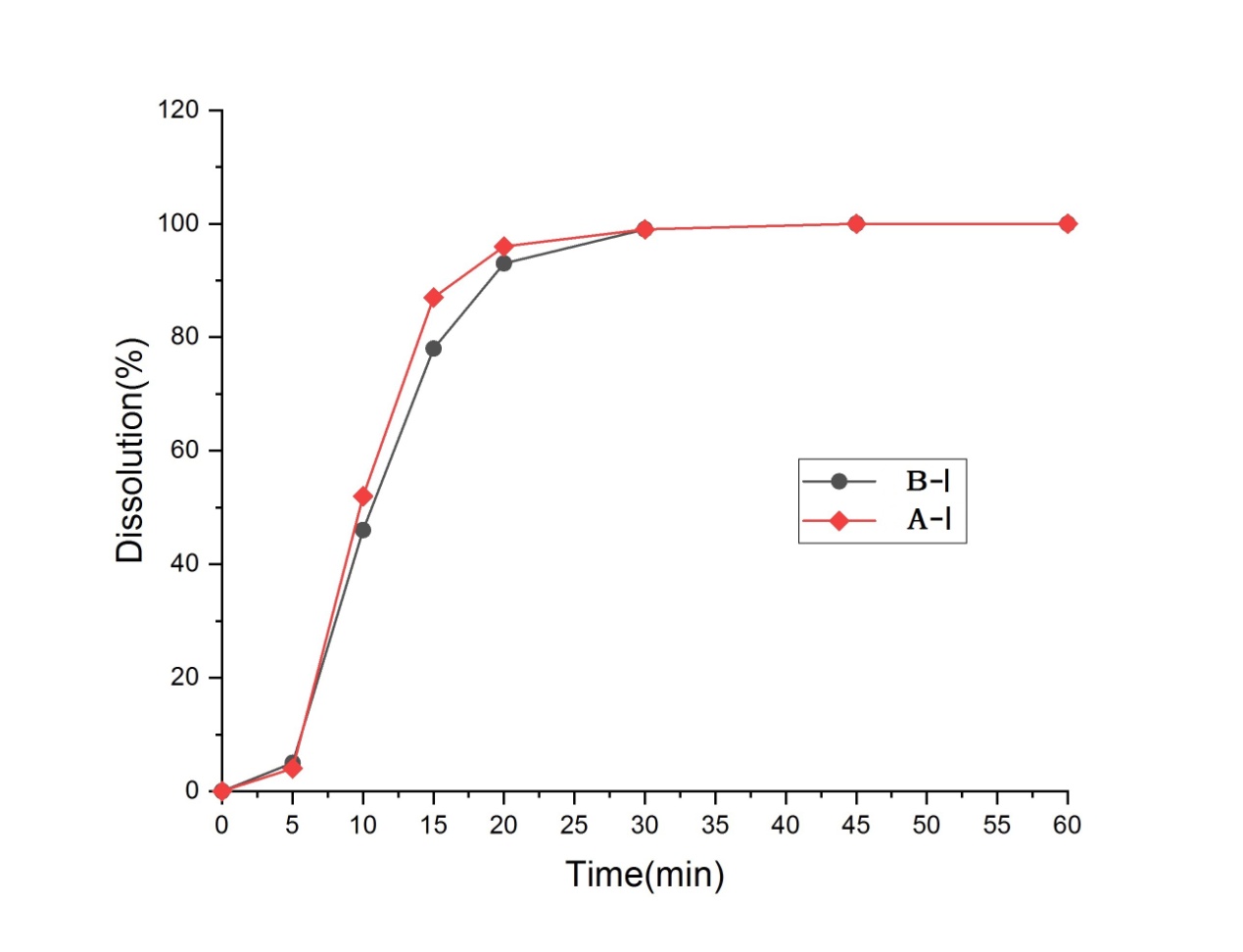


Fig.1 Dissolution profiles of A-1 and B-1 in pH6.8 medium.

Table 1. Mahalanobis distances between the dissolution curves of the samples from each batch.

| Samples | A-1 | A-2 | A-3 | A-5 | B-1 | B-2 | B-3 | B-4 |
| --- | --- | --- | --- | --- | --- | --- | --- | --- |
| A-1 | \ | 0.9428 | 1.3934 | 2.2181 | 3.0549 | 3.8192 | 2.5723 | 3.2437 |
| A-2 | 0.9428 | *\* | 0.9192 | 1.5582 | 4.2911 | 4.0235 | 3.5895 | 4.1657 |
| A-3 | 1.3934 | 0.9192 | \ | 1.7006 | 4.8262 | 4.9305 | 3.7915 | 4.5685 |
| A-5 | 2.2181 | 1.5582 | 1.7006 | \ | 5.2057 | 4.3077 | 4.3531 | 5.248 |
| B-1 | 3.0549 | 4.2911 | 4.8262 | 5.2057 | \ | 1.0263 | 0.2204 | 0.4797 |
| B-2 | 3.8192 | 4.0235 | 4.9305 | 4.3077 | 1.0263 | \ | 1.0692 | 1.9049 |
| B-3 | 2.5723 | 3.5895 | 3.7915 | 4.3531 | 0.2204 | 1.0692 | \ | 0.3899 |
| B-4 | 3.2437 | 4.1657 | 4.5685 | 5.248 | 0.4797 | 1.9049 | 0.3899 | \ |

Table 2. Standard deviation of the time entering PhaseⅢ for each batch of the six tablet samples.

| Samples | A-1 | A-2 | A-3 | A-5 | B-1 | B-2 | B-3 | B-4 |
| --- | --- | --- | --- | --- | --- | --- | --- | --- |
| RSD | 20.6% | 27.7% | 11.1% | 21.4% | 28.9% | 72.5% | 42.8% | 34.3% |
